# Heparins: A Shift of Paradigm

**DOI:** 10.3389/fmed.2019.00254

**Published:** 2019-11-15

**Authors:** H. Coenraad Hemker, Raed Al Dieri, Suzette Béguin

**Affiliations:** Department of Biochemistry, Cardiovascular Research Institute Maastricht (CARIM), Maastricht University, Maastricht, Netherlands

**Keywords:** heparin, low molecular weight heparin (LMWH), thrombin generation, endogenous thrombin potential (ETP), anti-thrombin activity, anti-factor Xa activity, activated partial thromboplastin time (aPTT), personalized medicine

## Abstract

Heparins inhibit the thrombin forming capacity of plasma, i. e., the endogenous thrombin potential (ETP), by their anti-thrombin (aIIa) activity, the anti-factor Xa (aXa) activity is of minimal importance. This holds for both unfractionated heparin (UFH) and low molecular weight heparin (LMWH) at aXa/aIIa ratios < 25. Clinical experience and epidemiological evidence show a direct relationship between the ETP and the risk of thrombosis and bleeding. Consequently, the therapeutic potency of a heparin is determined by its aIIa activity, i.e., the concentration of a domain in which 12 sugar flank the high affinity antithrombin-binding pentasaccharide (HA5) at one side. The response of individual plasmas to a fixed dose of any heparin is highly variable. This suggests that individualization of heparin dosage, on basis of the ETP, might reduce bleeding or re-thrombosis. There exist simple laboratory methods for both the ETP and the concentration of the active domain. These methods can be used both for unequivocally characterization of a heparin preparation and for controlling heparin therapy and allow arbitrary units relative to a standard to be abandoned. These tests are as robust as any hematological routine test but not yet routinely available, which severely encumbers progress in the field.

## Introduction

This article proposes nothing short of a paradigm change in heparin pharmacology. It shows that heparin acts by decreasing the amount of active thrombin formed in clotting (blood-) plasma, i.e., the endogenous thrombin potential (ETP) and that this activity is due to the anti-thrombin (aIIa) activity and not to the anti-factor Xa (aXa) activity. The activated partial thromboplastin time (aPTT), poorly reflects the amount of thrombin formed and therefore is poorly suited to reflect heparin activity.

Our argument is not new, it is based on a series of articles published in the last decades of the last century, to be referenced below. They went essentially unnoticed [see e.g., (1)], maybe because they relied on the ETP, the significance of which had not been firmly established until more recently. Below we will therefore first succinctly summarize the evidence that the ETP indeed is a trustworthy surrogate parameter of thrombotic- and bleeding-risk.

Then we will show what structure within a heparin molecule is responsible for the inhibition of the thrombin forming capacity and hence for its antithrombotic action.

Finally we will discuss how these insights may lead to an unequivocal characterization of heparin preparations, independent from arbitrary units and insensitive to adulterations such as those that caused the death of several patients in the first decade of this century (2, 3).

## Thrombin Generation Capacity Predicts Thrombotic and Bleeding Risk

In the last decades evidence has been accumulated which shows that the amount of thrombin-activity that forms in clotting blood correlates strongly with thrombotic- and bleeding risk.

The relation between high thrombin forming capacity and venous thrombosis has been established beyond reasonable doubt [e.g., by Hron et al. (4), Besser et al. (5), and Brummel-Ziedins et al. (6)]. That low thrombin forming capacity indicates a bleeding risk has been demonstrated as well (7–9). Congenital thrombotic tendencies all have an increased ETP and the acquired risk of thrombosis induced by oral contraceptives is quantitatively reflected in an increased ETP (10, 11). It has also been shown that high ETP and thrombotic tendency have a common genetic basis (12).

**Figure 1:**
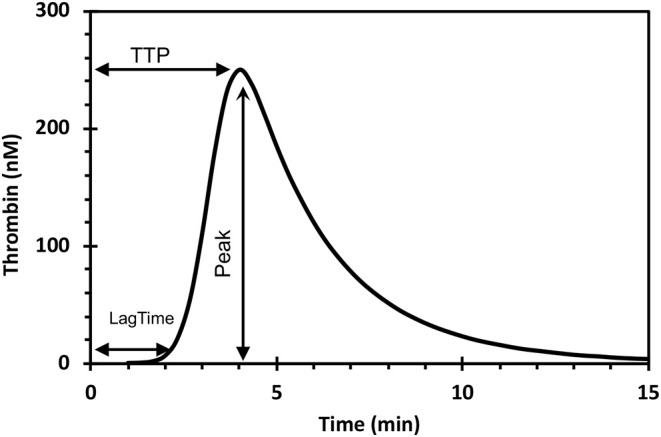
The thrombin generation curve and its parameters. The endogenous thrombin potential (ETP) is the area under the curve. TTP, time to peak.

The amount of thrombin that forms in clotting blood (-plasma) can be measured either as the peak- or the area under a thrombin generation (TG) curve (Figure 1).

The existing evidence can be summarized as:

*The more thrombin the more thrombosis but the less bleeding*,

*the less thrombin the more thrombosis but the more bleeding*.

TG-experiments are known for almost a century but became available for clinical- and pharmacological use with the advent of a semi-automated method (13, 14). The practical applicability has been delayed by problems of inter-laboratory repeatability but lately it has been shown that the experimental error is not worse than that of other coagulation-related tests (15, 16). For routine use different types of laboratory automatons have become available (17), but they are not yet in general use.

In the following text we will discuss the thrombin forming capacity mostly in terms of the area under the TG-curve, known as the endogenous thrombin potential (ETP). The gist of this variable is that it represents both the concentration of thrombin and the time that thrombin is active, i.e., the number of “man-hours” of enzymatic work that thrombin can de during its lifetime in plasma.

Thrombin generation is the physiological function that governs the plasmatic component of hemostasis and thrombosis. The antithrombotic action of anticoagulants lies in their power to diminish TG, even though their mechanisms of action may be totally different: AVK diminishes the concentration of prothrombin and other clotting factors, heparins enhance the decay of activated clotting factors and direct oral anticoagulants are pseudo-substrates that occupy the active center of activated clotting factors.

The potency of heparin preparations is therefore best quantified in terms of inhibition of thrombin generation. This allows us to broach the essential question of how heparins bring about this inhibition and what is the responsible active structure.

## Functional Anatomy of Heparins

Heparin is a long chain of sugar units that are negatively charged because they are substituted with acetic-acid and sulfate groups. For the understanding of heparin action in plasma it is not necessary to describe its chemical structure in more detail, except for one important feature: Sometimes in this chain there occurs a group of five sugars that are substituted in a specific way, so that they bind tightly to antithrombin^1^. This stretch of five sugars we will call the High Affinity Pentasaccharide (HA5). The presence of HA5 is a necessary—be it not sufficient—condition for the optimal activity of a heparin.

That there must be a tight-binding structure in part of the heparin molecules was found in 1976, independently by three groups. The teams of Lindahl (Stockholm) and Casu (Milan) determined the precise structure of this domain and Petitou, from the Choay team (Paris), synthesized it (1983). A detailed history of these discoveries is to be found elsewhere (18).

**Figure 2:**
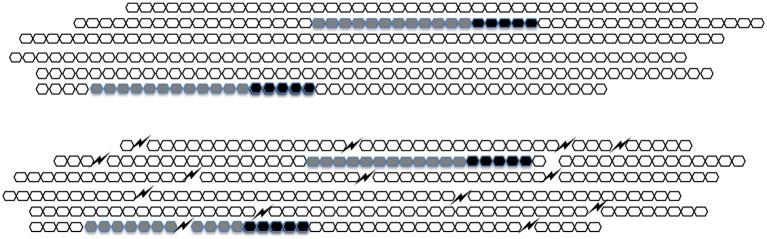
A schematic representation of heparin. In UFH (upper figure) every HA5 (black) has sufficient sugar moieties (gray) to its left side to form a C-domain. In LMWH (lower figure) the molecules are broken up and HA5 domains that do not belong to a C-domain appear.

In natural heparin HA5 occurs once in about every one-hundred sugar units. Upon extraction from its natural sources, heparin breaks up into pieces that typically fall in the range of 20–50 sugar units. About one in three molecules of unfractionated heparin therefore contains HA5 (Figure 2). When, during the preparation of low molecular weight heparins (LMWHs) unfractionated heparin is chopped up in smaller pieces the fraction of molecules that contain HA5 becomes lower and lower (Figure 2).

When antithrombin binds to HA5 its anti-FXa-activity increases considerably but its anti-thrombin-activity hardly does. For full anti-thrombin activity, it is necessary that HA5 is flanked by a stretch of 12 sugar units (of arbitrary structure) at its “left” (i.e., non-reducing) side. The combination of HA5 and the 12 sugar conveys full anti-thrombin activity to a heparin molecule. In honor of the late Jean Choay (1923–1993) who was the main driving force behind the development of LMWHs we called it the “Choay-domain” or C-domain.

## What is the Active Structure in Heparins?

Thrombin as well as factor Xa bind to plasmatic antithrombin to form an irreversible complex that has no enzymatic activity and that is cleared from the circulation. Heparins act by facilitating this process in two different ways:

In the first place, upon binding to HA5, antithrombin undergoes a conformational change that makes it slightly (∞ ~2x) more active toward thrombin and much more active against Factor Xa (~ 30x).

In the second place thrombin and factor Xa bind with low affinity anywhere on the heparin molecule. Once adsorbed, they will, by lateral, unidimensional diffusion (“sliding”), move toward HA5 and be inactivated there (19–21). To provide the “slide” for thrombin a minimum of 12 sugar units, i.e., the full C-domain is required.

Because thrombin binds some 10x more tightly to the heparin chain than factor Xa does, it profits much more from the “sliding” mechanism than factor Xa does (21).

In LMWH heparins there will be many HA5-domains that are no longer part of a C-domain (Figure 2) and that therefore have anti-factor Xa activity only. This causes the rise of aXa/aIIa ratio. It is a historical mistake to think that the increase of this ratio as such is responsible for the better clinical applicability of LMWHs over UFH. The difference is rather to be found in increased bioavailability [(22) see further below].

The aXa/aIIa ratio is anyhow overestimated because standard heparin, by definition, contains the same amount of aIIa units, and aXa units per mg. The biological activity, in terms of enzyme molecules inactivated per molecule of heparin per unit time, of UFH, and hence of the heparin standard, is three times higher toward thrombin than toward factor Xa. So in reality the aXa/aIIa ratio of UFH is 0.33 and all ratios mentioned for LMWHs are over-estimated by a factor 3. In fact, the situation is even more bizarre: aXa and aIIa activities are usually measured in the absence of Ca^++^ ions. When measured in presence of these ions—i.e., under plasmatic conditions—the ratios are again two times lower (23, 24).

## How do Heparins Inhibit the Thrombin Forming Capacity of Plasma?

**Figure 3:**
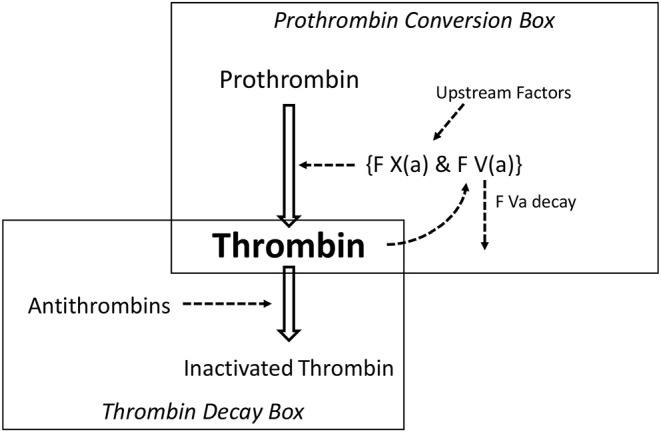
The minimal reaction scheme to explain a thrombin generation curve.

In Figure 3 we show a clotting scheme that suffices to explain the features of a thrombin generation curve (25). The reactions depicted in this scheme can be represented by a set of differential equations that render the course of thrombin as a function of time. The concentrations and reaction constants can be chosen such that any thrombin generation curve in the presence or absence of heparin can be exactly mimicked. We recall that prothrombin is converted into thrombin by a complex (prothrombinase) of factor Xa and factor Va. Clotting factors higher in the cascade exert their action by activating factor X. Factor V is activated by (meizo-) thrombin (positive feedback).

The system is triggered because, during the lag-time, tissue factor (TF) and factor VII activate an infinitesimal amount of factor X, that then produces tiny amounts of (meizo-)thrombin that activates factor V. Prothrombinase action is limited in time because the thrombomodulin-protein C system inactivates factor Va. These reactions occur at a membrane surface and therefore are not or hardly influenced by heparin, that is why the thromboplastin time (“prothrombin time”) is insensitive to heparins (Aside: the aPTT is heparin-dependant because the activation of factor VIII by thrombin takes place in free solution).

The model consists of a thrombin forming part, the prothrombin conversion box, and a thrombin scavenging part, the thrombin decay box.

We call this model the “washbasin” model because the concentration of thrombin is like the level of water in a washbasin—without stopper—in which a pail of water is quickly emptied. The level of the water will rise but as it rises the pressure on the outlet increases and the water will disappear with a velocity proportional to its level. When the pail is empty, the remaining water in the basin will disappear with a velocity proportional to its level and to the diameter of the outlet. If one knows the capacity of the outlet then, from the course of the level, it is easy to calculate the velocity with which the pail has been emptied.

Likewise, if the amount of antithrombin and the reaction constant of the association between thrombin and antithrombin is known, one can calculate the velocity with which prothrombin has been converted into thrombin (26, 27), i.e., one can calculate the activity of the prothrombin conversion box (Figure 2).

The surprising result of these calculations was that heparins appeared not or hardly to influence prothrombin conversion (28–30).

This can be explained by the fact that there is so much more factor Xa than factor Va, that one can afford important inhibition of FXa before this is felt as inhibition of prothrombinase. Originally it was thought that closing the tap (i.e., inhibiting FXa) would be more efficient than mopping the floor (inhibiting thrombin) (31). This idea is based on the cascade model of the clotting mechanism, in which one activated factor simply activates the next—nowadays it is realized that the thrombin generation mechanism is a finely tuned network, with positive and negative feedback loops. The cascade is the production line of the system but not the rate-determining feature.

That factor Va is indeed the rate limiting factor can be seen from the fact that the factor V_Leiden_ mutation, in which factor Va is longer-lived, indeed brings an increase of the ETP and a thrombotic tendency (32).

## Anti-Factor Xa Activity of Heparin Hardly Contributes to Heparin Action

That inhibition of factor Xa does not influence prothrombin activation is a far-reaching conclusion that requires further experimental confirmation. By column chromatography we separated heparin into fifty fractions of virtually single molecular weight and hence of single chain-length. Of each of the fractions we determined the concentration of the active pentasaccharide HA5 by fluorescence titration of purified antithrombin (33).

**Figure 4:**
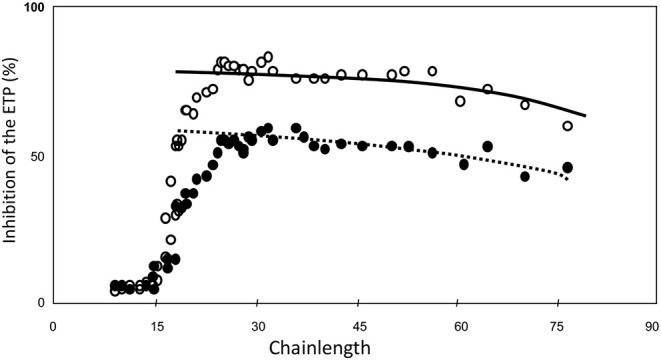
ETP inhibition as a function of heparin-chainlength. • 10 μM HA5-domain ° 25 μM. HA5 domain.

Then we tested the inhibitory power of each of the fractions when added at equal molar concentration of HA5 (Figure 4) (34). It is seen that the inhibitory power is critically dependent upon the chain-length (Figure 4).

Molecules that are too short to contain a HA5 + 12, i.e., a Choay domain and that therefore carry aXa-activity only are hardly inhibitory. It should be realized that in a natural heparin the HA5-domain is randomly distributed along the chain. A heparin molecule with its HA5 domain in the 11 leftmost positions will always carry aXa-activity only. The fraction of molecules with anti-thrombin activity therefore increases with the chain-length. If N is the number of sugar-units per molecule, then the number of positions for the HA5-domain is N-5, but for the Choay domain it is N-17. The concentration of C-domains therefore can be calculated from the concentration of HA5 if the chain-length is known, i.e., C = A.(N-17)/(N-5).

**Figure 5:**
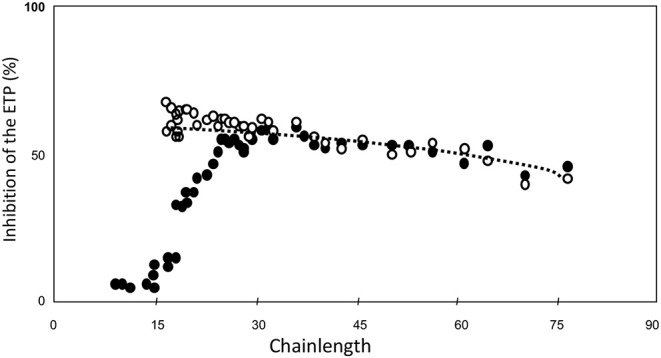
ETP inhibition as a function of heparin-chainlength. • 10 μM HA5-domain ° 10 μM Choay-domain.

If this calculation is carried out (Figure 5) we see that the inhibitory power is virtually constant per concentration of Choay-domain. The slight reduction with increase of chain-length can be attributed to (a) Increasing interaction with other plasma proteins than antithrombin and (b) Increasing probability that a thrombin molecule adsorbed on the chain dissociates from the chain before it reaches the HA5 domain.

We concluded that for any type of heparin, the capacity to inhibit the coagulation process in plasma is primarily determined by the concentration of Choay-domain, i.e., the AT-binding pentasaccharide with 12 or more sugar units at its non-reducing end.

## Why are LMWHs so Useful then?

The next question is: If LMWHs contain so little active material, why are they clinically superior to UFH? In our opinion this is primarily because of their superior bioavailability.

Bioavailability can be defined as: How much inhibition do I obtain per unit of heparin, and, as clinical dosage is usually done in terms of aXa-units: How much inhibition does one obtain from a fixed number of aXa-units?

**Figure 6:**
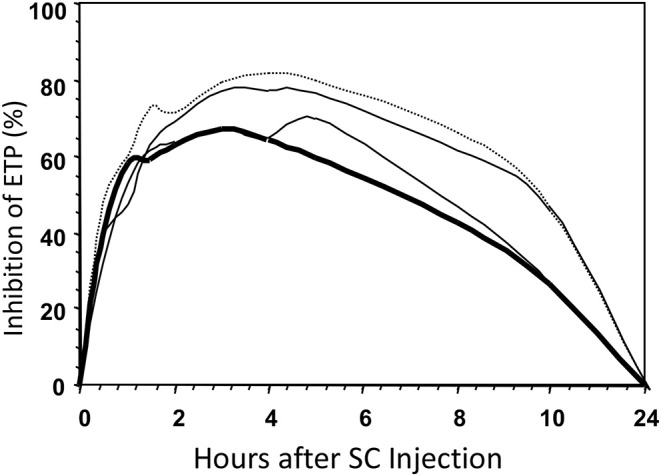
Inhibition of the ETP after SC injections of four different types of heparin in a volunteer. Bold line, UFH; dotted line, LMW with average MW of 4.5 kD; other lines, intermediate MWs.

To answer this question we injected 9,000 aXa-units of four different heparins into 12 volunteers (22) and determined the course of inhibition of the thrombin forming potential (Figure 6). The inhibitory effect of the injection was quantified as the area under this curve and the areas were normalized to that of UFH. The results are shown in Table 1.

**Table 1:** Bioavailability as a function of average molecular weight.

**Heparin**	**Avrg MW (kD)**	**aXa (U)**	**aIIa (U)**	**Availability (%)**	**Approx. half life (h)**	**CV (%) of Response**
**UFH**	13	9,000	9,000	100	0.5–1.5	31
**MMW**	10.5	9,000	8,100	140	2.5	17
**LMWH**	6.2	9,000	6,300	190	3.5	14
**ELMW**	4.5	9,000	4,500	340	4.5	17

It is obvious that bioavailability increases with decreasing molecular weight, so that, for the extreme low MW heparin e.g., only half the amount of active material (aIIa-units) is injected, but, compared to UFH, more than three times of this material becomes available for inhibition of the thrombin forming power of the plasma.

## The Merits of the Activated Partial Thromboplastin Time (aPTT)

Since its first description by Rappaport in 1961 (35) the aPTT has served many purposes, among which its use for quantifying the effect of heparins. Its utility for that purpose is widely doubted, however (1, 36).

We can add to this discussion that in the above mentioned experiment with 12 human volunteers (22) we injected four different heparins in 12 subjects at 9 time points between 0.5 and 10 h after injection. From the 432 samples that should contain heparin indeed 95% showed a significant aXa-activity but only in 34% the aPTT was significantly prolonged. The thrombin generation capacity (ETP) was significantly inhibited in 83% of the samples. This clearly shows the insufficiency of the aPTT.

Often the aXa-activity is suggested as an alternative [e.g., (36)] for the aPTT. This is not a useful suggestion, however. In the first place because it co-estimates a lot of irrelevant molecules, i.e., those with aXa-activity only. In the second place because the measurement of a concentration cannot replace the measurement of the pharmacological effect. We will see below that he same concentration of heparin in different plasmas will indeed have a very different inhibitory result.

## The Large Variability of the Heparin Effect

The common practice that LMWH administration is given at standard doses is based on clinical trials that show that in this way lung-embolism can be prevented and bleeding is not increased to an unacceptable degree [for references see (18)]. This does not answer the question whether results would improve when doses would be personalized.

It will be clear from the above that this question cannot be answered by measuring aPTT nor by estimating anti-factor Xa activity but the ETP serves its purpose here.

**Figure 7:**
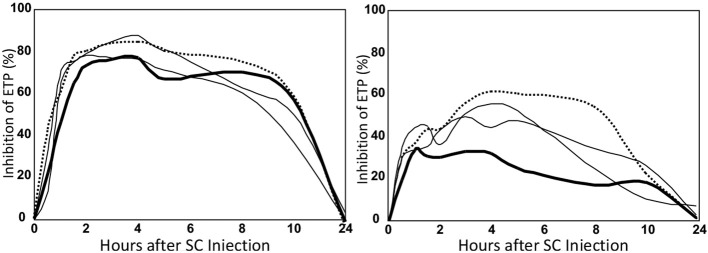
Inhibition profiles of a high **(left)** and a low **(right)** responder. Legend as in Figure 5.

In the abovementioned study (22) we also determined the variability of the over-all anticoagulant response between the 12 healthy volunteers (Figure 7 and Table 1, last column). It appeared that there was a very large difference in anticoagulated response.

In the highest responder an about two times higher anticoagulant effect was obtained as in the lowest responder (Figure 7). The variability was only partly explained by the variation in body-weight.

We also measured the inhibitory effect of 0.08 U/ml of UFH added to the individual plasma from 44 healthy donors and found a coefficient of variation of the inhibitory effect of 16%. Bloemen et al. (37), so the high variability is not only due to variations in availability of the drug but also to a variable responsiveness of the individual clotting system to the inhibitory action of the drug.

An as yet unpublished study [Table 2, cited by courtesy of Drs. A. Selmeszi (Debrecen) and R. Kremers (Maastricht)] shows that the uninhibited ETP already varies enormously between individuals (22–23%), but that the effect of a fixed concentration of heparin added to the individual samples causes residual ETPs that, especially in patients, vary so much as to become unpredictable.

**Table 2:** The variation of the inhibitory effect of heparins in different plasmas.

		**ETP (%)**	**CV-total (%)**	**CV-inhibition (%)**
Volunteers (*n* = 20)	Control	100	22	0
	UFH	53	33	25
	LMWH	40	35	21
	HA5	65	43	33
Patients (*n* = 30)	Control	100	23	0
	LMWH	47	57	52

This large variation in the individual heparin response shows that on a standard dose of any heparin many patients must be over- or under-treated. That there is nevertheless a well-defined beneficial effect on thrombosis (-prevention) shows that there must be a significant latitude between the risk of (re-)thrombosis or bleeding and the actual manifestation of these complications. This is nothing new: mild hemophilia can go unnoticed until middle age and congenital antithrombin deficiency will not show up until in the late teens.

In view of the large variability of response, we surmise that personalization of heparin dosage could considerably reduce the risks of heparin treatment, that of re-thrombosis as well as that of bleeding.

The question is whether current practice can be significantly ameliorated by personalized dosage. We surmise it would, but the cost-benefit relation remains an open question.

In conclusion: If one wants to control heparin pharmacokinetics, use aIIa activity, if one wants to know about pharmacodynamics, use thrombin generation.

## How to Characterize a Heparin Preparation

Heparin activities are still expressed in aIIa- and aXa-units relative to a standard, often using clotting times (aPTT) to establish the equivalence. When Howell discovered heparin around 1926 this was the only possible modus operandi but in this century, it is hopelessly outdated. Notably, because the aPTT is also sensitive to inhibition by polyanions that activate heparin cofactor II (HCII). Therefore, the aPTT—as prescribed by the pharmacopeias, does not detect adulterated heparins, with disastrous consequences (3, 38).

There is a simple and unequivocal manner to determine heparin activity in terms of standard independent, SI-based units (39). One standard independent aIIa-unit (SIU-IIa) of heparin is that amount of heparin that increases the decay constant of thrombin by 1 min^−1^ per μM of antithrombin. A SIU-Xa is defined analogously.

We described a very simple end-point assay to determine these decay constants (40). Because the assay uses a solution of purified antithrombin it will not co-estimate HCII-dependent contaminants. Such contaminants can be quantified by a similar test using heparin cofactor II instead of antithrombin.

The concentration of high affinity moieties (HA5) can be determined by fluorescence titration (33).

As a consequence, of any heparin preparation one can determine the activity in standard independent units per mole of high affinity material.

If one, for convenience, wants to use a standard, than any heparin preparation with a chain-length distribution of between 30 and 45 sugar units (100–150 kD) can be used, because in the range the inhibitory power is for all practical purposes constant per high-affinity molecule (Figure 4).

## The Ideal Heparin

The ideal heparin is the lowest molecular weight heparin that has a good inhibitory potency, i.e., a chain-length of around 25 units (around 8–10 kD) (Figure 4). The longer the heparin the shorter the half life time and the lower the bioavailability. The best heparin therefore presumably has MW-distribution of 10–20 kD.

The pure Choay domain, i.e., high affinity pentasaccharide with 12 sugar units to its non-reducing end would probably make the ideal heparin. Present day synthetic efforts still focus on aXa-activity (41–43) and attain a chain length of 12 sugar residues. It is clear, in the light of the above that a pure Choay-domain would be over 10-fold more effective than molecules with anti-factor Xa activity only.

## The Ideal Heparin Dosing

In view of the large interindividual differences in heparin responsiveness heparin dosage should be personalized so as to obtain an ETP of around 50% of the average in the normal normal population, i.e., 600–700 nM min (16). We hope that this paper might lead to rounding up experts in the field in order to start and perform a collaborative study that proves the suitability of ETP for this purpose.

## Author Contributions

HH wrote the first draft. SB and RA commented on it and the final text is the result of several amelioration loops.

### Conflict of Interest

The authors declare that the research was conducted in the absence of any commercial or financial relationships that could be construed as a potential conflict of interest.

## Footnotes

^1^There are many proteins in plasma (serpins) that can inactivate thrombin, antithrombin (formerly called antithrombin 3) is the main one, scavenging about two thirds of all formed thrombin.
